# Endoscopic transcecal appendectomy for recurrent appendicitis after previous endoscopic mucosal resection

**DOI:** 10.1055/a-2173-7608

**Published:** 2023-10-06

**Authors:** Zhi-Lan Ma, Hai-Ting Pan, Jia-Qi Xu, Ping-Hong Zhou, Zhen Huang

**Affiliations:** 1 Endoscopy Center, Taixing People’s Hospital, Jiangsu, China; 2 Endoscopy Center and Endoscopy Research Institute, Zhongshan Hospital, Fudan University, Shanghai, China

A 47-year-old man suffered from acute appendicitis 4 years ago, relieved by a 3-day course of oral levofloxacin. A follow-up colonoscopy 1 year later revealed a 1.5-cm submucosal bulge near the appendiceal orifice. The patient underwent endoscopic mucosal resection (EMR) for the lesion at that hospital ( Fig. 1 ) and was referred to our hospital for routine follow-up owing to the possibility of recurrence. Colonoscopy again found a submucosal bulge near the appendiceal orifice with purulent exudates ( Fig. 2 ).

**Fig. 1:**
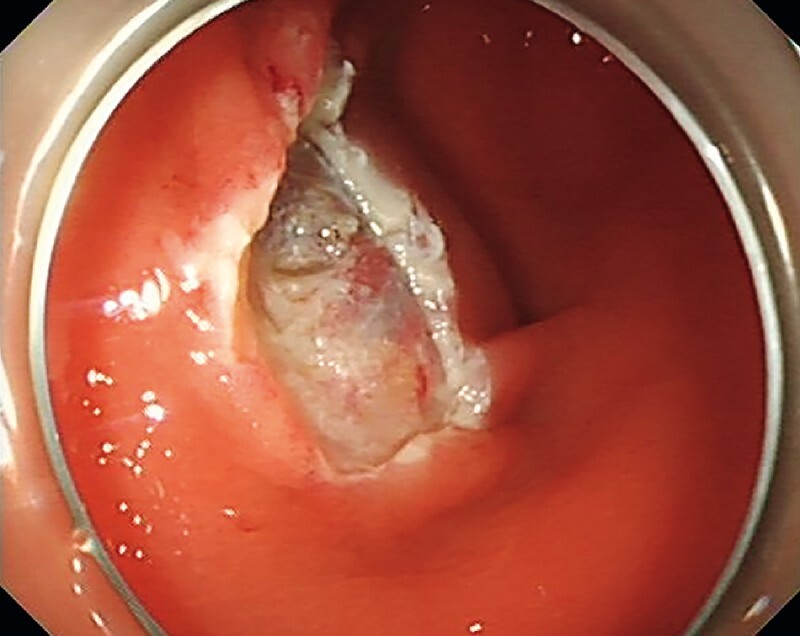
Previous endoscopic mucosal resection performed for the lesion.

**Fig. 2:**
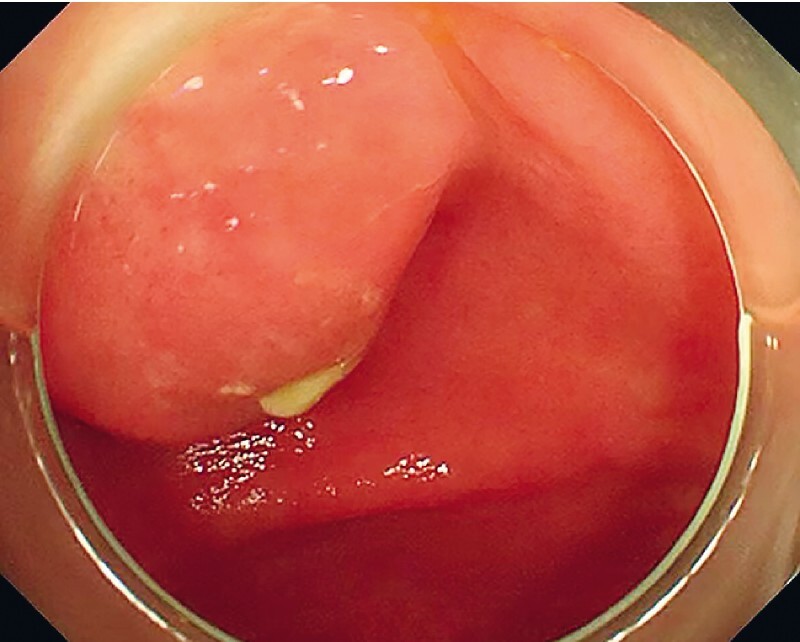
Colonoscopy found a submucosal bulge near the appendiceal orifice with purulent exudates.

Since the lesion was recurrent and simple drainage did not solve the problem, endoscopic transcecal appendectomy was suggested. A full-thickness resection was performed using an ITknife2 and a HookKnife, with submucosal injection and circumferential submucosal incision ( Video 1 ). The extent of resection was determined around the bulging appendiceal orifice. Subsequently, the endoscope was advanced into the peritoneal cavity, where the appendix was separated from the mesoappendix ( Fig. 3 ). The detached pelvic mesoappendix showed no active bleeding. After complete resection, the appendix was extracted into the colon and retrieved through the anus using a snare. The cecal wall defect was closed using the purse-string suture technique with seven endoclips and a nylon loop ( Fig. 4 ). The total duration time was 50 minutes.

**Video 1** Endoscopic transcecal appendectomy for recurrent appendicitis after previous endoscopic mucosal resection.


**Fig. 3:**
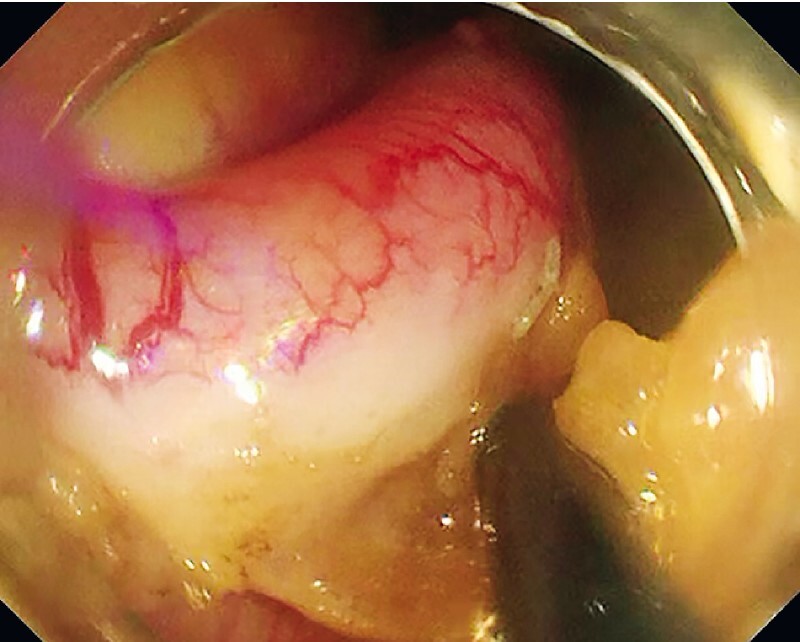
Separation of the appendix from the mesoappendix.

**Fig. 4:**
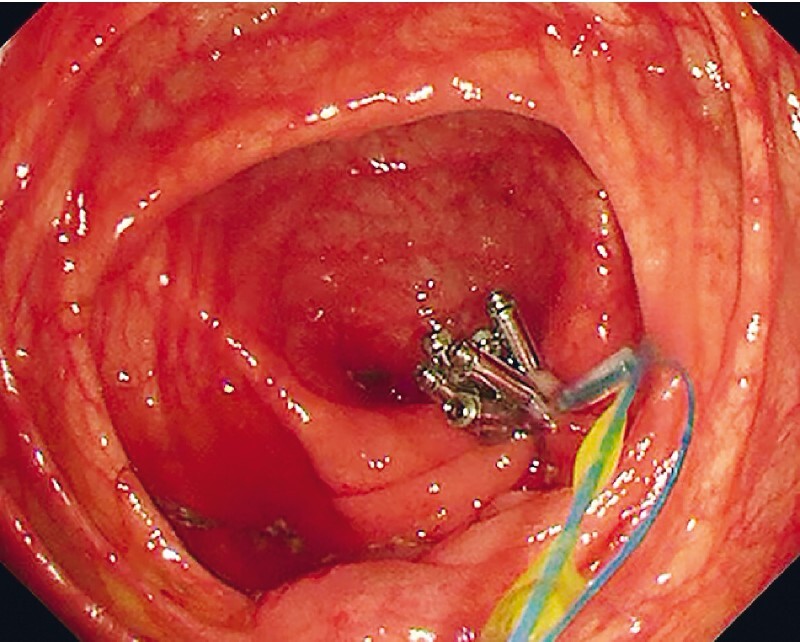
Closure of the cecal wall defect using the purse-string suture technique with seven endoclips and a nylon loop.

Pathologic diagnosis confirmed chronic appendicitis ( Fig. 5 ). The patient was discharged on postoperative day 5 without any complications. Natural orifice transluminal endoscopic surgery (NOTES) is emerging as a promising technique in the field of endoscopy. In this case, we have demonstrated that an endoscopic transcecal appendectomy in patients with previous EMR could be achieved successfully and safely. In the future, NOTES may be a promising option for chronic and recurrent appendicitis, challenging traditional or laparoscopic surgery as the first-line therapies for appendicitis. Therefore, further clinical studies and long-term follow-ups are necessary to validate the feasibility and effectiveness of NOTES in the treatment of appendiceal diseases.

**Fig. 5:**
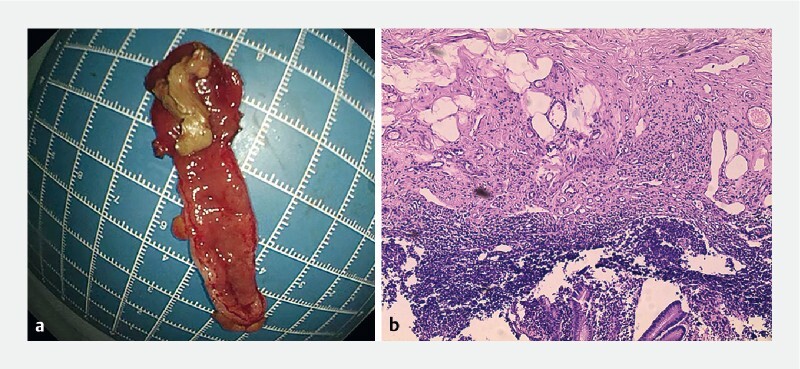
Complete resection of the appendix. Pathologic diagnosis confirmed chronic appendicitis.

Endoscopy_UCTN_Code_TTT_1AQ_2AC

## Notes

Endoscopy E-Videos

https://eref.thieme.de/e-videos
*E-Videos* is an open access online section of the journal *Endoscopy* , reporting on interesting cases and new techniques in gastroenterological endoscopy. All papers include a high-quality video and are published with a Creative Commons CC-BY license. Endoscopy E-Videos qualify for HINARI discounts and waivers and eligibility is automatically checked during the submission process. We grant 100% waivers to articles whose corresponding authors are based in Group A countries and 50% waivers to those who are based in Group B countries as classified by Research4Life (see: https://www.research4life.org/access/eligibility/ ). This section has its own submission website at https://mc.manuscriptcentral.com/e-videos

